# Credibility Assessment of the Patient-Specific Modeling of the Aneurysmal Ascending Thoracic Aorta: Verification, Validation and Uncertainty Quantification

**DOI:** 10.1007/s13239-025-00801-1

**Published:** 2025-08-28

**Authors:** Roberta Scuoppo, Chiara Catalano, Fabrizio Crascì, Salvatore Pasta

**Affiliations:** 1 Department of Engineering, Università degli Studi di Palermo, Viale delle Scienze Ed.8, Palermo, Italy; 2 Department of Research, IRCCS ISMETT via Tricomi, Palermo, Italy

**Keywords:** Uncertainty quantification and validation, ASME VV40; aneurysm of ascending aorta, Finite-element analysis, Computational fluid dynamics

## Abstract

**Purpose:**

Computational modeling holds promise in predicting patient-specific outcomes and guiding clinical decision-making. The patient-specific model forming the basis of a digital twin can be considered biomedical software, thereby necessitating trust in its predictive accuracy.

**Methods:**

This study applies the ASME V&V40 framework to demonstrate the credibility of patient-specific models of aneurysmal thoracic ascending aorta (ATAA) biomechanics. A comprehensive verification, validation, and uncertainty quantification process was performed to evaluate the accuracy of the patient-specific ATAA model.

**Results:**

After implementing the ASME V&V40 standard, the verification errors on the model inputs (i.e., material parameters and hemodynamic variables) resulted in relative errors (RE) < 1%. Validation and its uncertainty quantification of the output aneurysm diameter response showed area metric errors below 5% in the majority of cases, highlighting the accuracy of the patient-specific ATAA model against the clinical comparator. Uncertainties in wall stress predictions due to model inputs were also quantified by probability density functions. Sensitivity analysis revealed that the unknown value of aneurysm wall thickness drives the model output at the highest extent.

**Conclusions:**

These findings contribute to a standardized methodology for evaluating the credibility of patient-specific models, enhancing their utility in computer-based clinical decision support systems for managing patients with ATAAs.

**Supplementary Information:**

The online version contains supplementary material available at 10.1007/s13239-025-00801-1.

## Introduction

Computational modeling for biomedical products has gained significant attention as a viable alternative to replace and reduce in-vivo experimentation and bench tests. While there is evidence of modeling and simulation being utilized in various applications for obtaining market approval of medical devices, the widespread adoption of computational modeling as a standard tool is still being evaluated [1]. This evaluation extends not only to the regulatory framework governing medical devices but also to predictive patient-specific models, whether used independently or as part of medical device evaluation. Patient-specific modeling forms the cornerstone for digital twins, which serve as clinical decision-support systems for physicians to interpret and treat each individual patient. From a regulatory standpoint, these digital twins are classified as software as a medical device and must undergo certification for their predictive accuracy. This process is known as model credibility to quantify the in-silico methodology through evidence. Credibility for a computational model is not solely defined by its ability to replicate reality within predefined tolerances [2] but also takes into account the associated risks with the model outcome and its impact on decision-making [3].

The credibility of a computational model is established through a process of verification, validation, and uncertainty quantification, which aims to demonstrate its accuracy and reliability. An applicability assessment, including model risk and the rigor of output comparison, is also part of the credibility assessment. Verification involves determining whether a computational model accurately represents the underlying mathematical model and its solution. Validation assesses the extent to which the model accurately represents corresponding physical experiments or in-vivo data, considering the intended uses of the model. Uncertainty quantification involves characterizing uncertainties in the model and its inputs, considering population variability, and establishing the impact of inputs parameter variations in model outputs. Finally, model risk encompasses the potential for the computer model to produce incorrect results, leading to undesirable outcomes in subsequent decisions. Recently, FDA guidelines [1] have proposed a generalized framework for assessing model credibility, primarily based on the American Society of Mechanical Engineers (ASME) V&V40 “Verification and Validation in Computational Modeling of Medical Devices” [3]. No standard is currently available for credibility assessment in the EU regulatory system. The use of ASME V&V40 has also been observed in computational modeling for drug development [4], demonstrating the flexibility of this technical standard across various fields.

This study focuses on applying the ASME V&V40 framework to assess the credibility of computational modeling, specifically targeting a patient-specific model of the structural biomechanics of the aneurysmal thoracic ascending aorta (ATAA). Patient-specific ATAA modeling was chosen as a case study because of its potential to predict wall stress for prognostic purposes, contrasting with the limited predictive capability of aortic size criteria [5]. Although wall stress analysis in ATAAs was investigated for predicting the rupture risk in bicuspid and tricuspid patients [6, 7], this manuscript primarily addresses the credibility assessment of patient-specific modeling.

## Methods

The overall study design involved (i) constructing finite-element models based on patient-specific CTA imaging and biomechanical inputs, (ii) verifying the model accuracy through discretization and numerical solver analysis, (iii) validating model predictions of aneurysm diameter against clinical measurements by probabilistic assessment, and (iv) quantifying uncertainty in both diameter and wall stress predictions via surrogate modeling and quasi-Monte Carlo simulations.

### Standard Description and Application

The ASME V&V40 framework was utilized to establish the credibility of the patient-specific ATAA model [3]. The process initiates with defining the Question of Interest (QoI). Subsequently, the Context of Use (CoU) and associated model risk assessment were delineated to guide the establishment and specification of credibility goals and the rigor of the assessment.

The QoI is formulated as follows: “Can the patient-specific ATAA model replicate the function of the aneurysmal aorta during cardiac beating?” From a biomechanical standpoint, the severity of ATAA relies on factors such as weakened material tissue, heart rate, blood pressure, and structural loading.

For the CoU, considering the significant variability in patient demographics, physiological conditions, and material tissue properties, the use of patient-specific modeling to simulate physiopathology facilitates the prediction of the aneurysm diameter.

Model risk encompasses the potential for the computer model to produce an incorrect result (i.e., the model’s influence) and for a subsequent decision based on the model to lead to an undesirable outcome (i.e., the model’s consequence). Regarding model influence, the simulation output from the computational model significantly influences the decision-making process, thus necessitating a high level of rigor. As for model consequence, even though the model is not directly used to inform decisions regarding a medical device, which could potentially lead to severe patient injury, we deemed it necessary to maintain a high level of credibility. Hence, a severity level of 5 for model credibility was chosen in the development and validation of the patient-specific ATAA model, following the 5-level risk matrix outlined in ASME V&V40.

The so-established severity level served not only to quantify the rigor of the necessary verification but also the validation and uncertainty quantification. Regarding output comparison rigor, a severity level of 5 indicates that comparisons, along with uncertainty estimates, between the comparator (i.e., the medical imaging) and the computational model are based on differences of ≤ 5%.

### Patient-Specific ATAA Model

The finite-element model of the aneurysmal aorta was constructed starting from segmentation performed using the medical imaging software Mimics (v21, Materialise, BE) [8]. Semi-automatic thresholding of the diastolic phase of electrocardiogram-gated computed tomography angiography (CTA) images was utilized to obtain the lumen of the aneurysmal aorta (ethical approval IRRB/04/04). While a comprehensive credibility assessment should include quantification of thresholding uncertainties, the fidelity of the reconstruction process was not investigated in this study.

Following segmentation, unstructured finite-element grids were generated using ICEM meshing software (v2021, Ansys Inc, PA, USA) [9]. The biomechanical response of the ATAA wall was modeled using a 2nd order form of Ogden’s constitutive relationship, with material parameters extrapolated from biaxial data reported by Azadani et al. [10]. Though the aorta has an anisotropic behavior, the stress-strain curve reported by Azadani and collaborators shows a slight loss of mechanical directional dependency due to histopathological changes as seen in other ex-vivo studies of the aneurysmal aorta [11, 12]. Thus, the stress-strain curves (*n* = 20) reported by Azadani et al. [10] were fitted for both the circumferential and longitudinal directions of each patient tissue using the fitting tool Hyperfit (v2.1, Czech Science Foundation). This allowed to extract a representative range of Ogden’s material parameters suitable for modeling aneurysmal tissue behavior. To guarantee the stability of simulations, the combinations of material parameters from the fitting output leading to invalid material response (µ1 + µ2 > 0, µ1α1 > 0 and µ2α2 > 0) were excluded. Material thickness was considered to be uniform throughout the aorta, despite variations in the sinuses and circumferential vessel direction. Modeling the variations of material thickness and associated uncertainty was deemed difficult in the current study.

Simulations were performed using the finite-element solver Abaqus (v2023hf2, Dassault Systemes, FR) with a quasi-static mechanics formulation and explicit solver. The ATAA wall was subjected to a physiological pressure waveform, as previously described [13]. Specifically, systolic and diastolic values of the pressure waveform were scaled according to the investigated blood pressure bounds (refer to Table 1). The heartbeat was used to scale the duration of the pressure waveform and set the simulation time. The assumption of uniform pressure distribution over the whole aorta is a restriction since pressure pulse propagation and spatial variation might influence aneurysm propagation. However, predicting the uncertainty of non-uniform pressure distribution in the aorta wall may be regarded as challenging. Prior to the simulation of the cardiac cycle, the zero-pressure configuration of each ATAA was obtained using the approach developed by Krishnan et al. [14] for the aneurysmal aorta. In brief, the ATAA wall was assumed to have a supra-physiological stiffness and was loaded at a diastolic pressure of 80 mmHg. This condition aimed to generate a stress distribution on the loaded vessel without causing geometry deformation. Then, the supra-physiological stiff material model was replaced by the true material properties and deflated from diastolic pressure to 0 mmHg, thereby determining the zero-pressure unloaded geometry. The simulation time to obtain the zero-pressure configuration was one second, with the timesteps automatically chosen by the numerical solver in Abaqus/Explicit. For boundary conditions, the distal ends of the descending aorta and supra-aortic vessels were fixed in the longitudinal vessel direction using cylindrical coordinate systems. The aortic annulus was protruded 2-fold the annulus diameter, and then both downward stretch and twist were applied at the proximal end of the extrusion to account for heart-induced motion of the aneurysmal aorta. For each patient, systolic CTA images were utilized to compute the stretch and twist with respect to the diastolic vessel configuration. Specifically, longitudinal displacement of the aortic annulus between systole and diastole was calculated to estimate axial stretch. The twisting motion was quantified by measuring the angular change of the sinus of Valsalva between the two phases, utilizing the sinus bulge as a reference landmark. Viscous pressure was incorporated to enhance numerical stability and dampen out low-frequency dynamic effects of the explicit formulation. Similarly, Rayleigh damping was employed to account for the effect of explicit formulation, resulting in numerical damping in the form of bulk viscosity. The cardiac beat simulation involved initially pressurizing the zero-pressure configuration to diastolic pressure using a linear ramp, followed by the simulation of two cardiac cycles. Simulations were performed on a server with two AMD EPYC 75F3 processors, enabling the simultaneous execution of two jobs, each utilizing 30 CPUs. The mean simulation duration was 0.52 ± 0.25 h, with variations attributed to discrepancies in mesh size, material parameters, or boundary conditions among models.

### Verification

Verification aimed to quantify computational errors arising from the numerical representation of ATAA physiopathology. Verification activities were performed to address discretization error (DE), numerical solver error (NSE), and numerical code verification (NCV). These errors must be kept negligible to ensure they do not compromise the quality and rigor of the validation and uncertainty quantification processes. A relative error (RE) < 1%, which was computed as the normalized difference between reference and test values, was adopted as the verification criterium for all error sources. A list of bugs and errors known to be present in the computational code was reviewed and assessed as part of software quality assurance. Errors associated with user input were not included in the analysis.

For DE, mesh refinement and element formulation were explored to estimate discretization estimates. Three mesh sizes were tested (0.8 mm, 0.6 mm, 0.4 mm), and the resulting aneurysm diameter at peak systole was used as the output quantity. For NSE, solver settings such as damping coefficients, mass scaling, and viscous pressure were varied individually while keeping the mesh constant. The reference solution was defined using default solver parameters, and RE was calculated on the aneurysm diameter. For NCV, a representative benchmark problem was identified for the patient-specific ATAA model, and this benchmark was compared against analytical solutions. Specifically, the problem of a thin-walled cylindrical vessel under internal pressure was modeled, and the computed circumferential stress was compared with the analytical value given by LaPlace’s law ( $\:\sigma\:=P\bullet\:r/2\bullet\:t$) where $\:P$ is a reference pressure of 120 mmHg, $\:r$ is radius of 10 mm) and $\:t$ is the thickness of 2 mm. The RE between numerical and analytical stress was computed to assess the model’s ability to accurately solve the underlying mathematical equations governing vessel biomechanics.

### Validation

Validation aimed to estimate the prediction error and associated uncertainty of the patient-specific ATAA model. The definition of model inputs and outputs is fundamental for validation activities. Model inputs include patient demographics (i.e., blood pressure and heartrate), material models, and boundary conditions, while the model output is represented by the aneurysm diameter. Evaluation of the model inputs and outputs for the various quantities of interest defined in the QoI must be conducted in comparison to a comparator.

#### ATAA Model Input

The model inputs considered for sensitivity analysis included constitutive material parameters, aortic tissue thickness, heart rate, systolic pressure, diastolic pressure, and flow velocity across the aortic valve. After assuming these quantities as epistemic uncertainties, we computed the nominal values along with the lower and upper bounds for each model input (refer to Table 1).

**Table 1:** Nominal values as well as lower (LB) and upper (UB) bounds of each model inputs. All parameters were considered as epistemic variables

	nominal	LB (-2s)	UB (+ 2s)
**mu1** (MPa)	5.0	-0.01	20
**a1** (-)	7.3	-29.4	45.1
**mu2** (MPa)	-0.4	-2.9	0.10
**a2** (-)	18.8	-98.8	14.6
**s** (mm)	2.0	1.2	2.9
**HR** (bpm)	70.0	51.0	100.0
**Psys** (mmHg)	120.0	95.0	180.0
**Pdias** (mmHg)	80.0	60.0	101.0
**V** (m/s)	1.3	0.8	4.0

Note: s = thickness; HR = heart rate; Psys = systolic pressure; Pdias = diastolic pressure; v = transaortic flow velocity

The bounds for Ogden material parameters were determined through the fitting of the biaxial stress-strain response of the aneurysmal aorta, as described in Sect. 2.1. Conversely, the bounds for blood pressure and heart rate were extrapolated from a large clinical database developed in our hospital institution [15]. Tissue thickness of the aneurysmal aorta was derived from ex-vivo data reported previously by our group [16, 17].

#### Comparator

As a comparator, computed tomography angiography (CTA) was employed to quantify the aneurysm diameter at peak systole. Aortic diameter was chosen as the validation output because of its clinical significance and recognized relevance in guiding surgical decision-making for patients with ATAAs. Understanding the range of characteristics of the sample is essential to define the applicability and validity of the proposed patient-specific ATAA model. In this study, a test sample of six patients with ATAAs, comprising three with bicuspid (BAV) and three with tricuspid aortic (TAV) valves, was considered. The range of characteristics of the test sample included patient age (34–82 years), cuff systolic pressure (110–152 mmHg), cuff diastolic pressure (68–80 mmHg), heart rate (63–75 bpm), and flow velocity across the aortic valve (1.1–2.1 m/s). A one-to-one comparison of the aneurysm diameter versus the CTA diameter measurement was performed as the measurement of the test sample. Given the resolution of the CTA scanner at 0.488 × 0.488 × 0.625 mm, an uncertainty up to 0.625 mm on the clinically-measured aneurysm diameter was accounted for the validation assessment. Three measurements of the aortic diameter were carried out by the same operator.

### Quasi-Monte Carlo and Surrogate Modeling

The validation assessment concludes with the quantification of uncertainties in model outputs distribution resulting from the uncertainties of the model inputs. It was possible to carry out a sensitivity analysis to assess how uncertainty in outputs can be divided and allocated to different sources of uncertainty which takes into consideration the probability distribution functions (PDFs) of the input parameters. Validation relied on the initial development and subsequent use of a surrogate model for model response evaluation in uncertainty quantification, due to the high computational cost associated with quasi-Monte Carlo analysis. The use of surrogate modeling was also motivated not only by computational cost, but also by the necessity to execute efficient and robust uncertainty quantification across a multidimensional input space. Evaluating high-fidelity finite-element simulations for each input sample in a quasi-Monte Carlo setup would be computationally prohibitively expensive, especially when modeling several patient-specific anatomies with nonlinear material behavior.

**Fig. 1:**
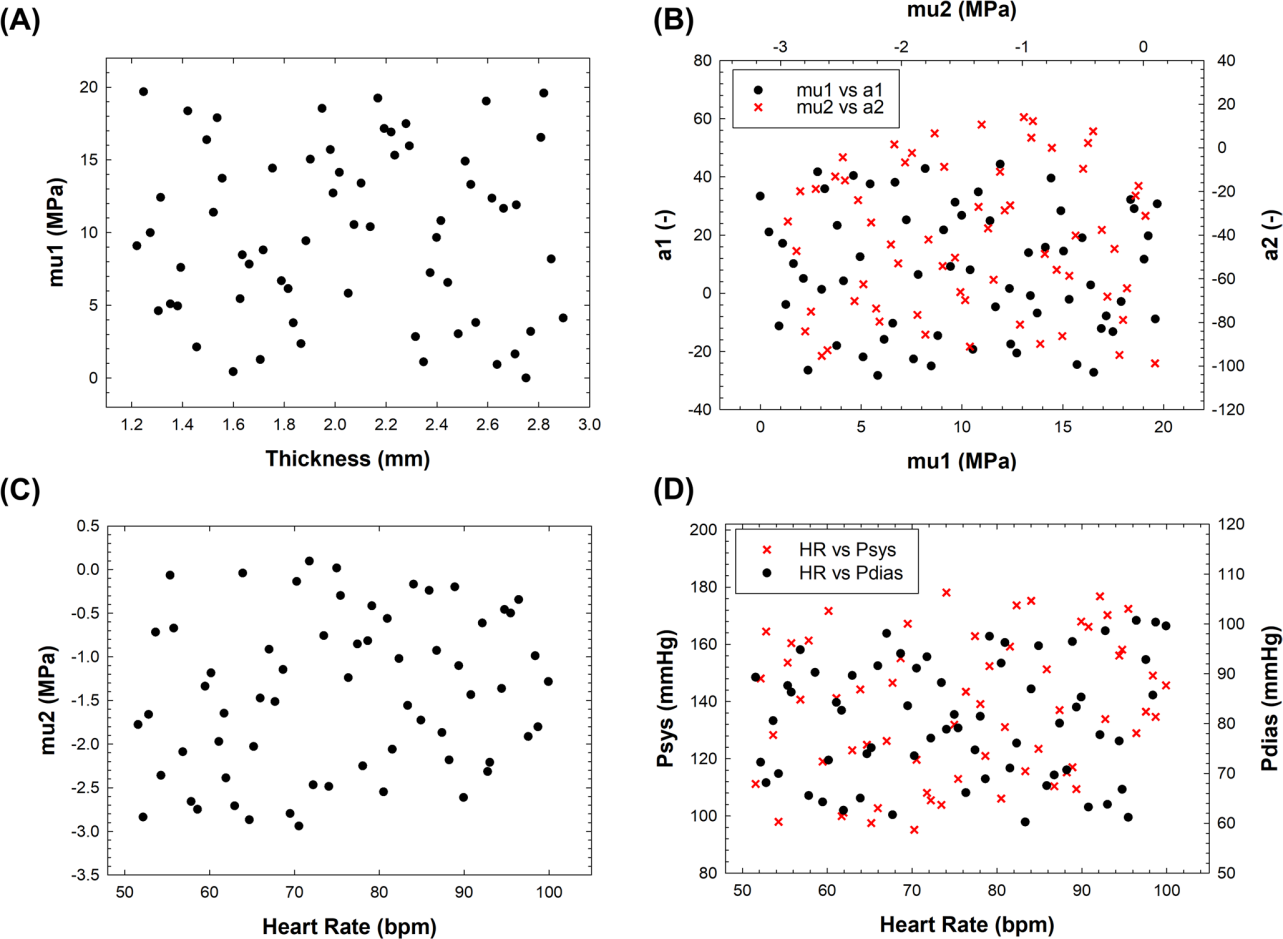
Latin hypercube sampling of model inputs showing interaction of (**A**) the tissue thickness versus 1st shear modulus; (**B**) four material descriptors; (**C**) 2nd shear modulus versus heart rate; (**D**) heart rate with diastolic and systolic pressure

The surrogate model was developed using Latin hypercube sampling to generate a finite-element simulation plan comprising 9 × 7 random combinations of input variables (number of input variables x number of samples), as shown in Table 1. For each patient, 63 FE simulations were therefore performed to develop the surrogate model. Once trained and validated, the surrogate model was used to generate 10,000 predictions as part of the quasi-Monte Carlo approach. Representative interactions among input parameters are shown in Fig. 1.

After each finite-element simulation, the aneurysm diameter at peak systole was extrapolated using a Python script. This involved initially computing the circumference described by three points at the mid-height of the ATAAs and then determining the circumference diameter. The surrogate model was constructed using Gaussian process regression with leave-one-out cross-validation to approximate the response of the original finite-element model at a relatively low computational cost. In the leave-one-out cross-validation, each of the 63 simulations was excluded once from the training set and its corresponding output predicted by the surrogate built from the remaining 62 simulations. The prediction error was then calculated for each of these left-out points to determine surrogate model performance.

Sobol sequences were generated for quasi-Monte Carlo simulation to sample the input space, with the samples scaled to fit specified input variable ranges. The surrogate model replaced the original finite-element simulations to generate deterministic data points for the Sobol approach. Sensitivity analysis was performed using Pareto plots to identify which input parameter variations most contribute to the uncertainty in model outputs. The total effect indices (TEIs) was used to express the percentage influence of each input parameter on the total output variance of Pareto plots. For validation assessment, the cumulative density function (CDF) of the surrogate-derived aneurysm diameter was computed. Similarly, the CDF was also determined for the comparator, assuming a normal distribution of the aneurysm diameter measurement with specified mean and standard deviation (i.e., the patient aortic diameter and the uncertainty given by the scanner accuracy). The area under the model and comparator CDFs was calculated (namely, the area metric), and the percentage difference between them was determined to assess the model’s accuracy.

### Uncertainty on Wall Stress

An additional quasi-Monte Carlo investigation was carried out to explore the sensitivity and uncertainties of the stress distribution in the patient-specific ATAA model due to variance in model inputs (e.g., material parameters). For each patient, a new surrogate model was trained using input simulation data to predict the maximum principal stress at the mid-height of the aneurysmal aorta. Subsequently, Pareto plots and probability density plots were assessed from the quasi-Monte Carlo analysis to evaluate the sensitivity and uncertainties associated with the wall stress predictions.

## Results

### Verification Analysis

**Fig. 2:**
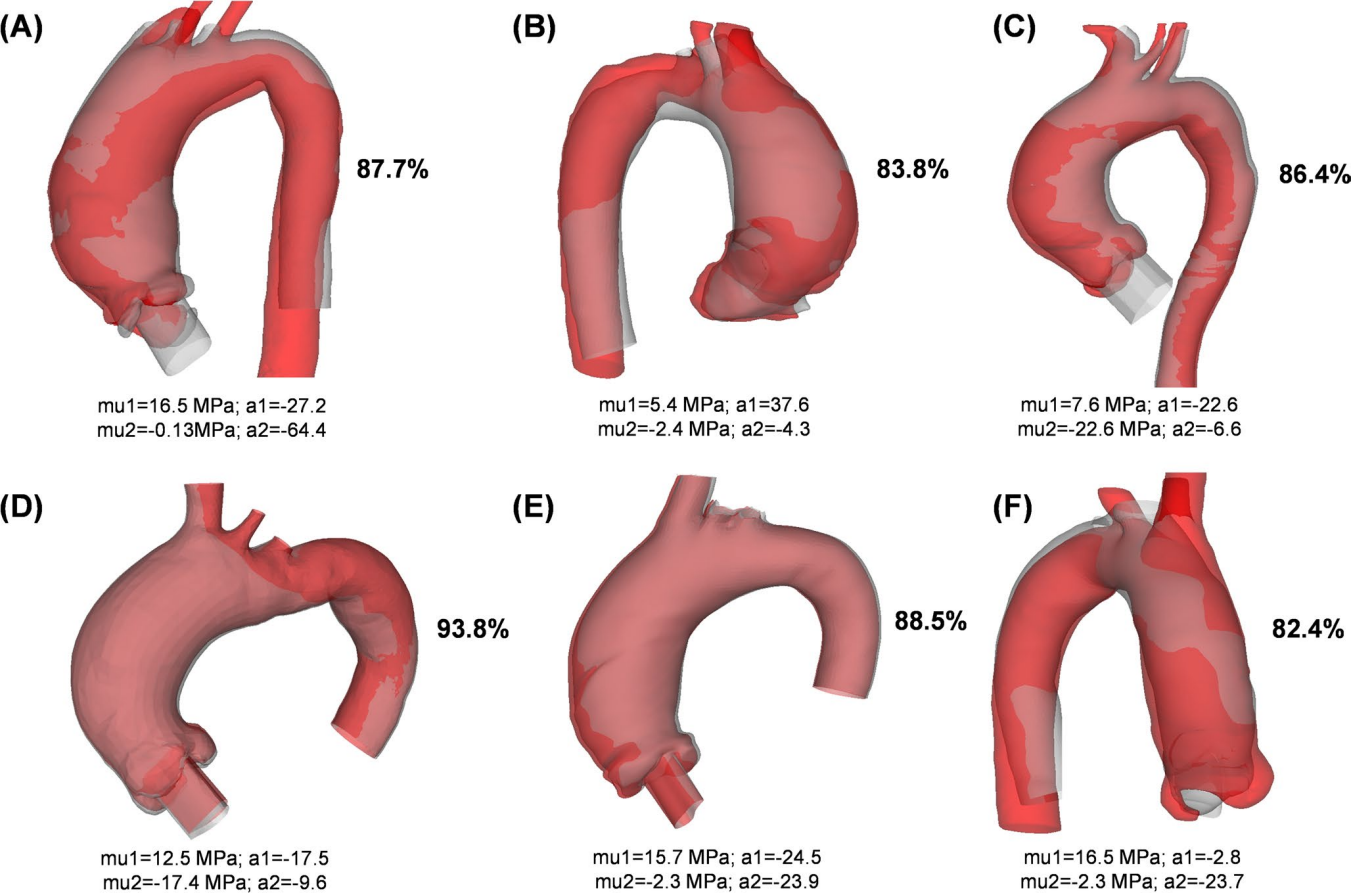
Comparison between numerical predictions (grey color) and actual CTA-related (red color) shapes of ATAAs at peak systole for all patient models (A-F); the IoU is shown in percentage, and the material parameters are also shown

To determine the level of agreement between numerically predicted and medical imaging data, systolic CTA scans were segmented at peak systole and compared to numerical predictions established at various model input values. To assess shape consistency, the intersection over union (IoU) was employed to determine how well the predicted ATAA shapes overlap with the true CTA-related shapes at systole. Figure 2 depicts a visual examination of agreement between numerically-predicted and actual CTA-related ATAA shapes. The analysis demonstrated a good agreement between computationally-predicted and actual ATAA shapes (IoU in the range of 82%-93%), with differences mostly detected in the region of high curvature or the supra-aortic vessel.

Verification analysis of the patient-specific ATAA model resulted in negligible errors in the structural simulation development (Table 2). The element size and solver settings that yielded a RE < 1% for both DE and NSE were retained for subsequent validation and uncertainty quantifications. Thus, the element size of 0.6 mm was adopted for all uncertainty and validation activities, with the aortic wall having damping factor of 50 and viscous pressure of 2.0e-5. Similarly, the optimal mass scaling was 1.0e-5. The use of viscous pressure and Rayleigh damping with compared to simulation without these parameters proved helpful in reaching numerical convergence in simulation situations with high heart rate while without altering the simulation result. Regarding solver setting For NCV, the benchmark problem of a thin hollow cylinder under uniform pressure and small deformation was employed. The numerical analysis of the cylindrical vessel utilized mesh size and solver settings resulting from the DE and NCV analyses, respectively. The model was subjected to an arbitrary pressure load of 120 mmHg, and the resulting circumferential stress was used as the output parameter. Upon solution, a RE of 0.6% was observed between the benchmark model and the analytical solution derived from the LaPlace law.

**Table 2:** Verification activities performed for the model ATAA #1 showing RE for DE, NSE and NCV

Structural Simulation		RE (%)
**Discretization Error**	Size (mm)	
S3–0.8	0.8	5.7
S3–0.6	0.6	0.9
S3–0.4	0.4	/
S3R– 0.6	0.6	1.2
**Numerical Solver Error**	Value (1/s)	
damping 50	50	0.2
damping 100	100	0.4
damping 200	200	0.8
	Value (-)	
mass scaling 1.0e-5	1.0e-5	0.8
mass scaling 1.0e-6	1.0e-6	1.3
mass scaling 1.0e-7	1.0e-7	15.5
	Value (MPa)	
viscous pressure 2.0e-5	2.0e-5	0.8
viscous pressure 2.0e-6	2.0e-6	0.7
**Numerical Code Verification**	Value (kPa)	
LaPlace’s law	31.6	0.6

### Surrogate Modeling Performance

**Fig. 3:**
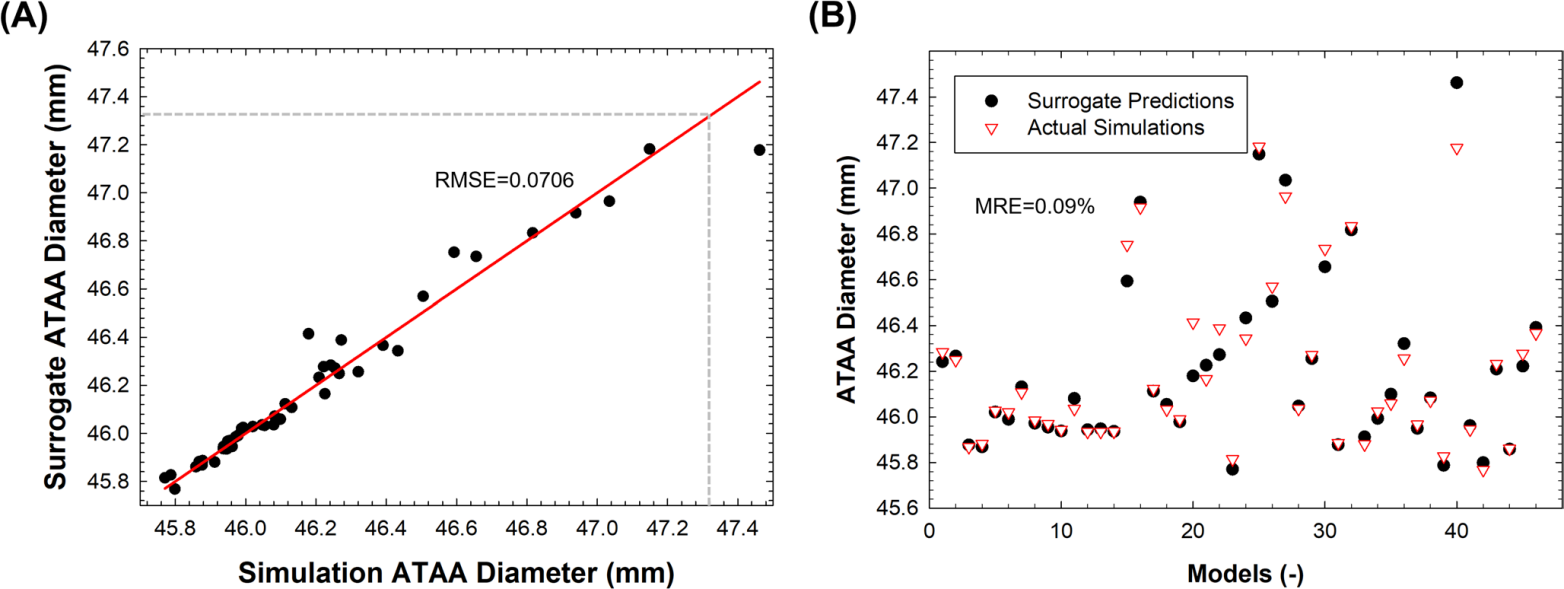
(**A**) correlation between the surrogate model response and actual finite-element predictions of aneurysm diameter for model ATAA #1; (**B**) plot of surrogate predictions and actual numerical simulations across the entire dataset of different simulations (x-axis) as generated by the design of experiment for the patient case ATAA #1

Out of the 63 finite-element simulations planned in the Latin hypercube sampling, only a small fraction of simulations failed due to unrealistic combinations of material parameters and pressure loads. Figure 3A illustrates the comparison between predictions made by the surrogate model and actual simulation values for one representative patient-specific ATAA model. The response of the trained surrogate model demonstrated great predictive capability in estimating the aneurysm diameter, as evidenced by low leave-one-out root mean squared error (LOO RMSE) and high correlation coefficients observed across all patients (see Table 3).

**Table 3:** Clinical parameters and surrogate model response across ATAAs in case of both aneurysm prediction and wall stress predictions against actual finite-element results

				ATAA Diameter			Max Princ Stress		
	D (mm)	Valve (-)	Age (yrs)	LOO RMSE	*R* ^2^ (%)	MRE (%)	LOO RMSE	*R* ^2^ (%)	MRE (%)
**ATAA #1**	47.2	TAV	57	0.076	0.98	0.1	0.022	0.99	9.2
**ATAA #2**	46.6	BAV	34	0.090	0.98	0.1	0.008	0.99	3.6
**ATAA #3**	54.6	BAV	53	0.102	0.99	0.1	0.004	0.99	1.2
**ATAA #4**	43.8	TAV	68	0.048	0.99	0.2	0.031	0.98	7.2
**ATAA #5**	47.7	BAV	61	0.059	0.99	0.1	0.011	0.99	2.6
**ATAA #6**	42.0	TAV	82	0.077	0.99	0.2	0.019	0.99	4.6

Note: D = ATAA diameter; LOO RMSE = leave-on-out root mean square error; R = correlation coefficient; MRE = mean relative error

In Fig. 3B, the plot of surrogate predictions and actual numerical simulations across the entire dataset is presented. Mean relative error (MRE) was utilized to quantify the average relative differences between surrogate and simulation-related values. The accuracy of predicted ATAA diameter was high, with MREs ≤ 0.2% observed for all patient-specific ATAA models. This is probably attributable to the surrogate model’s capacity to capture the non-linear response of the aortic wall, combined with the relatively limited anatomical variability of the included ATAA models and the simplicity of the physical problem addressed.

### Sensitivity Analysis

**Fig. 4:**
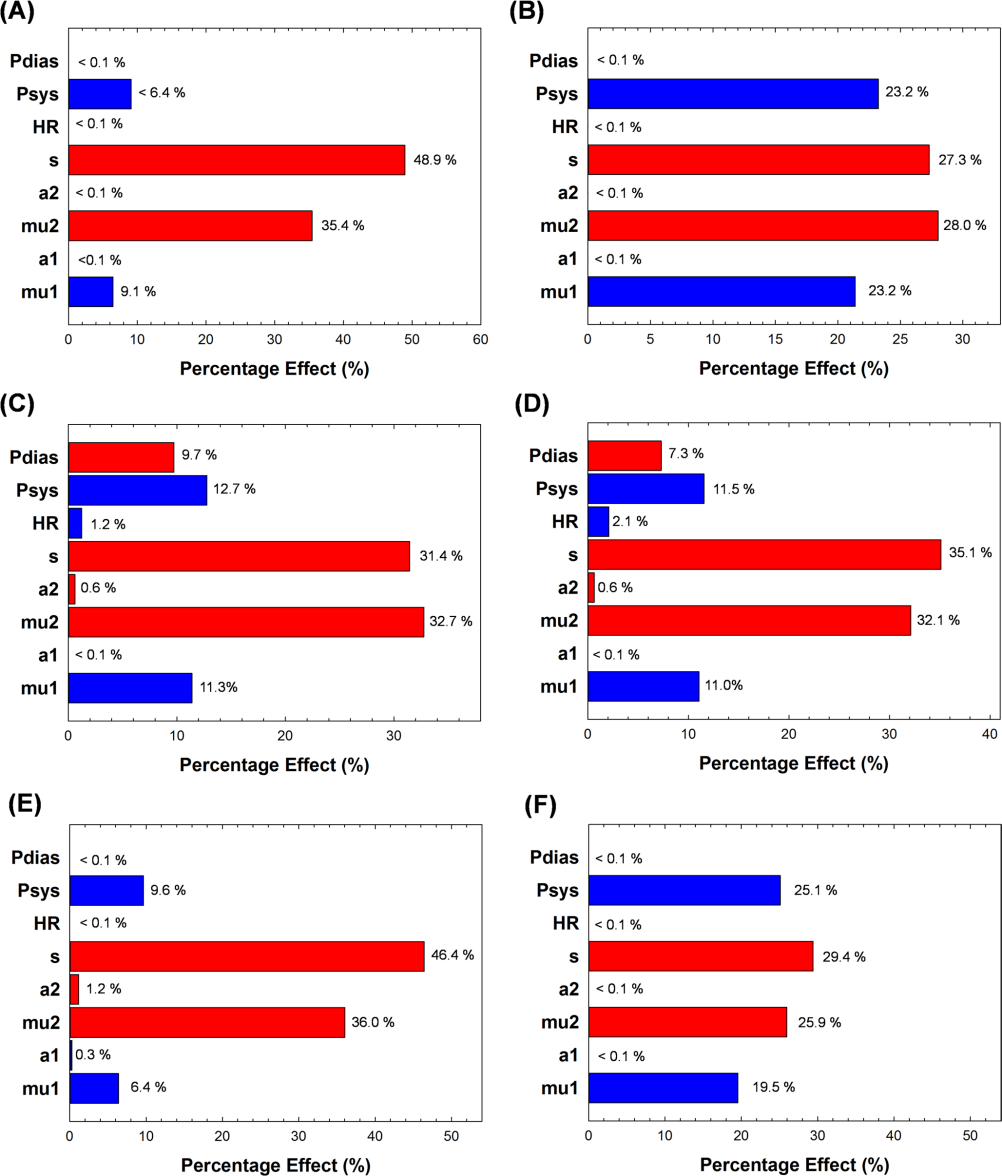
Pareto plot showing sensitivity of model inputs on the output diameter response for model ATAA#1 to #6 (**A**-**F**). The blue color indicates positive correlation whilst the red color indicates negative correlation

Figure 4 displays the Pareto plots of input model parameters on the diameter output uncertainties. Blue and red Pareto bars indicate whether the input variable is positively or negatively associated with the output variable, respectively. Sensitivity analysis revealed that the thickness of the ATAA wall is the most influential input parameter on the diameter output, with percentage effects ranging from 27.3 to 48.9%. The second most influential input parameter was the 2nd shear modulus (mu2), representing the stiffness of the material response, which emerged as the more significant among material descriptors. In contrast, patient-specific ATAA modeling showed lower sensitivity to changes in diastolic pressure, heart rate, and the Ogden’s parameter associated with material non-linearity (alpha1). Large changes in these parameters resulted in marginal effects, with percentages lower than 2.1% observed for all ATAA models. The variability in the influence of diastolic pressure across patients may be due to inter-subject differences in anatomical or biomechanical characteristics; however, the current sample size is insufficient to draw broad conclusions.

### Validation Analysis

**Fig. 5:**
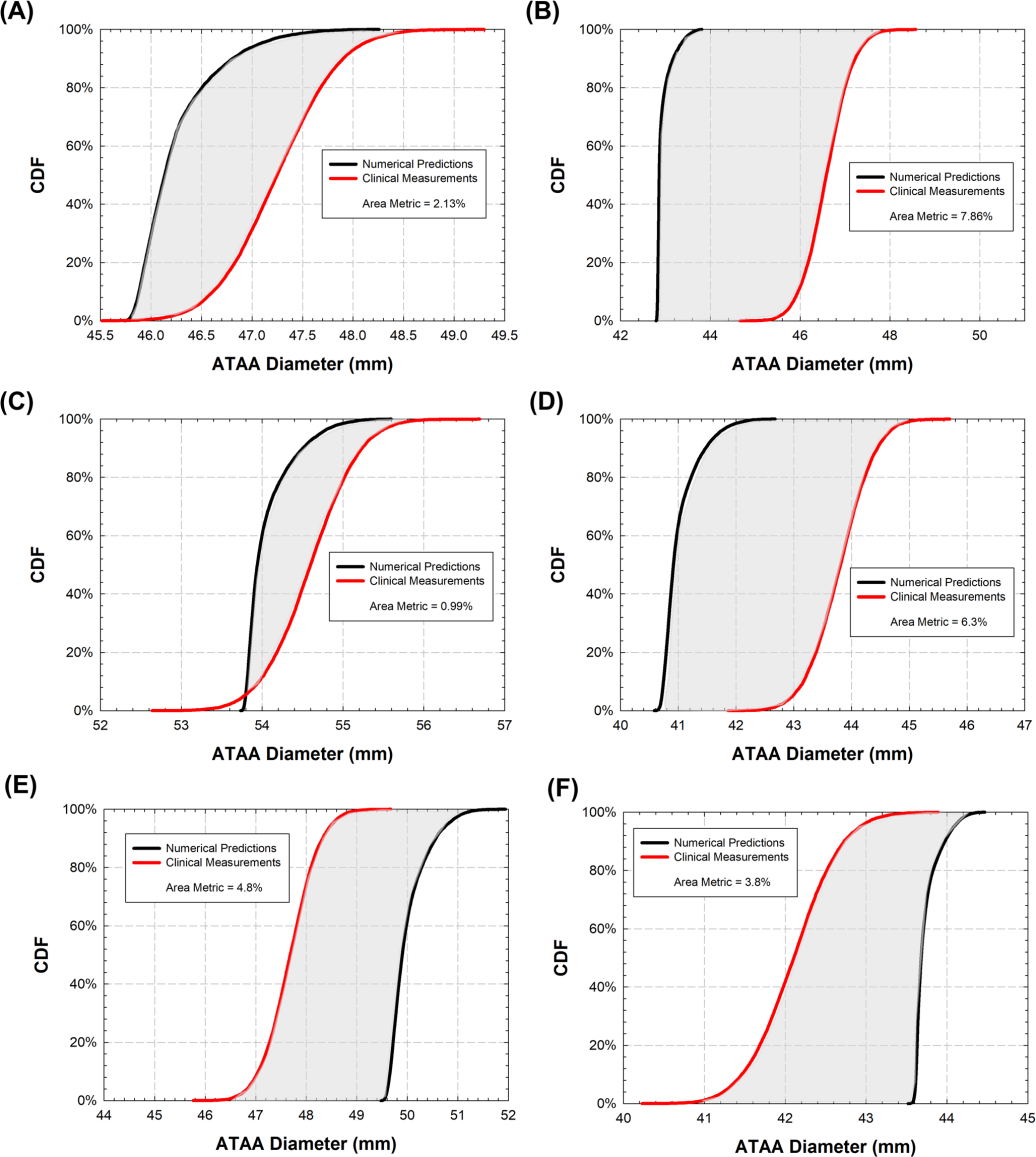
CDF curves for both the simulation and comparator showing the area metric confined by the two CDF curves (grey area) and the resulting validation metric for model ATAA#1 to #6 (**A**-**F**)

Figure 5 illustrates the cumulative density function (CDF) curves for both computational models and CTA-related measurements to assess the accuracy for model validation. A general trend was observed, with numerical simulations tending to underestimate the aneurysm diameter in four out of six patients and thus the biomechanical response of the vessel. This is evidenced by a leftward shift of model CDFs compared to those of the CTA-based comparator. For the BAV ATAA with aortic size of 46.6 mm (Fig. 5B), the variability of the predicted aneurysm diameter did not overlap with the CTA-related measurement and its uncertainty. Conversely, for the patient with TAV ATAA and size of 54.6 mm (Fig. 5C), the probability of having an aneurysm size ≤ 54.6 mm was 94% for the investigated input model variance. Most importantly, the area metric, serving as a quantitative parameter of the difference among the CDFs, was below the targeted 5% of established rigor of assessment for all patients except for the two BAV ATAAs.

### Uncertainty of Aneurysm Wall Stress

**Fig. 6:**
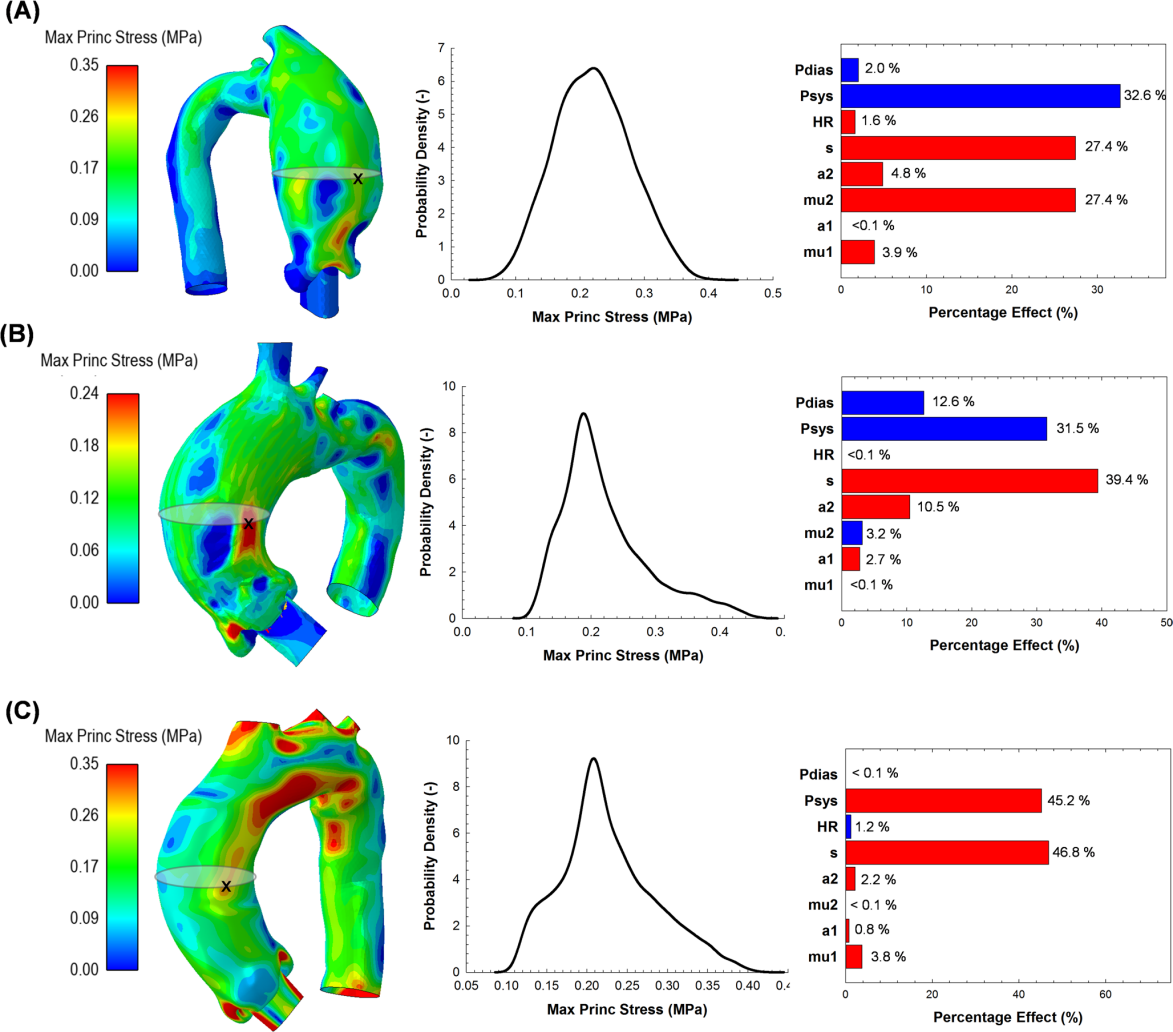
Uncertainty analysis on in-plane maximum principal stress showing PDFs and Pareto plots for TAV ATAA models with (**A**) diameter of 47.2 mm (ATAA #1), (**B**) diameter of 43.8 mm (ATAA #4) and (**C**) diameter of 42.0 mm (ATAA #6)

**Fig. 7:**
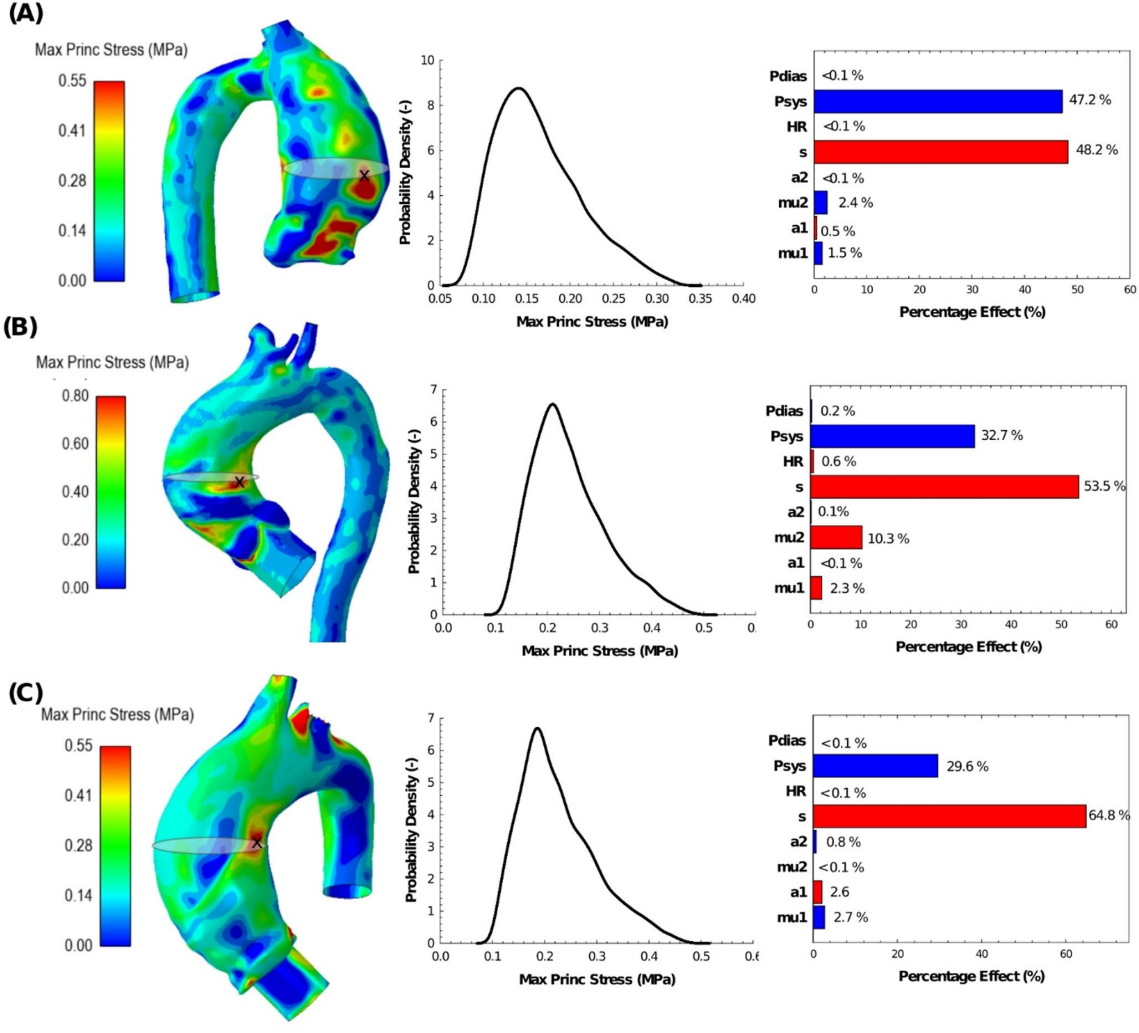
Uncertainty analysis on in-plane maximum principal stress showing PDFs and Pareto plots for BAV ATAA models with (**A**) diameter of 46.6 mm (ATAA #2), (**B**) diameter of 54.6 mm (ATAA #3) and (**C**) diameter of 47.7 mm (ATAA #5)

Once the validation assessment with uncertainty quantification was completed, the variance of resulting in-plane stress distribution on the patient-specific ATAA models was explored using probability density function (PDF) and Pareto plots for both TAV (Fig. 6) and BAV patients (Fig. 7). For each patient, the map of maximum principal stress is shown for the set of model inputs that provided a simulation aneurysm diameter closest to that observed in CTA images at systole. The PDF for each ATAA illustrates the uncertainty of the wall stress due to changes in material parameters and pressure loading conditions. The likelihood of wall stress of ~ 0.2 MPa is highest among patients. Moreover, stress maxima are primarily sensitive to peak systolic pressure and tissue thickness, with percentage effects ranging from 31.5 to 47.2% and 27.4–53.5%, respectively. Pareto plot color bars indicate that an increase in systolic pressure and a decrease in thickness can increase the resulting maximum principal stress. Table 2 presents the accuracy of the trained surrogate models in terms of LOO RMSE and MRE for predicting the uncertainty in aneurysm stress. It is worth noting that MREs between surrogate- and simulation-related stress values were less than 10% for all patient-specific ATAA models.

A detailed explanation for the grades assigned to each credibility activity is given for the verification and validation with its applicability to the COU (see supplementary material).

## Discussion

This study performed a rigorous credibility assessment of a computer model simulating the biomechanical behavior of the aneurysmal ascending aorta. The specific concern addressed by the patient-specific ATAA model was the quantification of aneurysm size (i.e., the QoI), followed by the definition of the role and scope of the model in relation to in-vivo CTA data (i.e., the CoU). Model influence was defined as the contribution of constitutive material parameters, aneurysm thickness, heart rate, and pressure loading on the model output diameter. Once the model risk requirements were established, the credibility assessment was completed through verification, validation, and uncertainty quantification, as dictated by ASME V&V40. Comparison and uncertainty estimates with differences ≤ 5% (i.e., severity level of 5) were achieved in four out of six ATAA models, indicating that patient variability may influence the quality of the credibility assessment. The uncertainty intrinsically presented in the unknown value of wall thickness assignment influenced the aneurysm size response to the highest extent. Findings suggested that the proposed patient-specific ATAA model has sufficient credibility to serve as a foundation for decision-making in alignment with regulatory guidelines for biomedical products.

Evaluating the accuracy of in-silico models is crucial for establishing the model’s reliability within a defined CoU. Several studies in various bioengineering fields, such as thrombectomy [18], thoracic endovascular repair [19], cardiac electrophysiology [20], left ventricle blood flow after LVAD [21], hemolysis [22], and femur fracture [23], have carried out reliability analyses of numerical models. To the best of our knowledge, this is the first study to focus on the credibility assessment of patient-specific ATAA modeling following a framework based on ASME V&V40.

Current clinical decision-making regarding the aneurysmal aorta’s risk stratification of adverse events still primarily relies on measuring the maximum aneurysm diameter through diagnostic imaging [24]. However, over the last decade, computational modeling and simulation have demonstrated the significance of stress-based risk assessment for aneurysm failure [7, 25]. Viewing the aorta as a material, the likelihood of rupture or dissection arises from an imbalance between aneurysm stress and strength. This concept has been incorporated into two commercial software packages, ViTAA and Vascopos, which offer a rupture potential index specifically-designed for abdominal aortic aneurysm failure. Before adopting a computational model for regulatory purposes, it is required to demonstrate the credibility of its predictions with evidence. Our goal was to practically apply the ASME V&V40 standard to perform a technical validation of the proposed computational model for patients with ATAAs. The assessment was successfully accomplished, as patient-specific ATAA models demonstrated a high level of accuracy against the comparator, with values of the area metric lower than the 5% criterion for rigor of assessment in four out of six patients. The fact that two patient-specific ATAA models had an area metric above the 5% acceptance criterion is likely due to our implicit assumption that all model inputs are affected solely by uncertainty due to measurement errors. In patient-specific modeling validation, it is crucial to recognize that model inputs are influenced not only by the individual phenotype but also by the broader patient population. Therefore, uncertainty should encompass inter-subject variability. Indeed, the ASME V&V40 standard does not explicitly address how to accommodate the unique characteristics of patient-specific modeling or integrate them into subsequent steps of the standard application. Apart from our current study, another significant exploration of ASME V&V40 with a focus on patient-specific model credibility is the research conducted by Galappaththige and colleagues [26] concerning patient-specific cardiac electrophysiological modeling. They highlighted that the accuracy of validation, and consequently the model’s accuracy concerning the QoI and CoU, is heavily dependent on the chosen validation approach.

Sensitivity analysis has provided valuable insights into the technical implementation of the computational modeling. Ensuring the accurate value of the thickness of the aneurysm wall is essential for the proper development of patient-specific ATAA models. This parameter holds paramount importance, as direct quantification from routine clinical imaging is not possible. Analysis of the total Sobol index highlights that tissue thickness ranks highest among the model inputs across the ATAA models. The chosen thickness significantly influences the diameter response, accounting for approximately 50% of the variation, which is more impactful than the selection of material parameters. In contrast, hemodynamic parameters were found to have less influence on assessing the biomechanical response of the aneurysmal aorta.

Validation involved comparing the predicted aneurysm diameter to the actual imaging diameter and its associated uncertainties. This method transitioned deterministic simulation output into a non-deterministic value, defined by the probability of observing a desired ATAA diameter. The adoption of ATAA diameter as the output parameter was evident, as there is currently no methodology to quantify wall stress in-vivo. Once the patient-specific ATAA model was validated, uncertainty quantification on wall stress revealed that variability in both aneurysm thickness and systolic pressure are the most critical factors influencing wall stress variability. A peak systolic ATAA wall stress of ~ 0.2 MPa is most probable, yet a considerable range of variability exists due to uncertain model inputs (ranging from 0.05 MPa to 0.4 MPa across all ATAA models). These findings are pertinent for designing new computational strategies to reduce uncertainty in wall stress predictions. While many research groups have focused on developing constitutive models [27] or calibrating material parameters [28], our findings suggest that more attention should be given to modeling structure of aortic wall thickness along the aneurysmal aorta. Ex-vivo biaxial material testing has shown variations in material properties with the thinner structure of the Sinus of Valsalva compared to the ascending aneurysmal aorta [29]. Surprisingly, none of the computational studies in the literature have modeled the ATAA as a variable wall.

This credibility assessment should be confined to the proposed implementation of the patient-specific ATAA model. A change from shell to solid aneurysm wall, the utilize of anisotropic material properties or the utilize of MRI as comparator implies a re-assessment of the credibility investigation. For the sake of simplicity, validation was performed on the same group of patient used for model development. However, validation should be performed ex-novo as part of the validation process performing a prospective investigation or retrospective analysis on new patient-specific models. It is also important to note that the clinical diameter measurements were performed by a single operator; the potential inter- and intra-observer variability was not quantified, which may have led to an underestimation of the total measurement uncertainty included in the comparator CDFs. The reader should consider that there may be a certain degree of interpretation or judgement of ASME V&V40 framework. For instance, the agreement of output comparison should be the predominant factor when compared to the credibility level, but the ASME V&V40 does not specify this aspect. Another question concerns the choice of an appropriate validation metric. The RE parameter with probability is only applicable in situations where there are clinical results and simulation results per sample. The advantage of the area metric is that different degrees of variance in the clinical and numerical results could be detected while the mean value remains identical. However, if the clinical and simulation do not intersect each other, then the area metric becomes insensitive to differences in variance. Another minor issue was related to the assessment of the output property in ASME V&V-40. While the test samples and test conditions need to be assessed with several credibility factors including measurements and measurement uncertainty, the actual system output used for comparison as part of the validation is not specifically assessed. The ASME V&V40 does not specify if output is also a test condition or not. While the ASME V&V40 framework defines patient-specific models as medical device software, the standard does not specify how to address variability in the validation activities across patients. The present findings are limited by the small cohort size, and broader generalization will require validation on a larger patient sample.

## Conclusions

We conclude that this study demonstrated the strength of the ASME V&V40 framework for assessing the computational modeling credibility of patient-specific ATAA models. Through rigorous verification, validation and uncertainty quantification process, we demonstrated the accuracy of our model with errors below 5% versus the comparator in the majority of cases. Our findings highlighted the importance of considering uncertainty in wall stress predictions and emphasized the impact of aneurysm thickness on stress variability or aneurysm size. This research contributes significantly to the field by providing a practical example for evaluating the credibility of patient-specific models, thus enhancing the potential of computer-based clinical decision support systems in managing patients with ATAAs.

## Electronic supplementary material

Below is the link to the electronic supplementary material.

Supplementary material 1

## Data Availability

The data that support the findings of this study are available from the corresponding author, SP, upon reasonable request.

## References

[1] (FDA) FaDA. Center for Devices and Radiological Health, Assessing the Credibility of Computational Modeling and Simulation in Medical Device Submissions,. https://www.fda.gov/media/154985/download; 2021.

[2] Schruben LW. Establishing the credibility of simulations. Simulation. 1980;34(101–105).

[3] (ASME) TASoME. Assessing Credibility of Computational Modeling Through Verification and Validation: Application to Medical Devices, ASME V&V 40-2018,. 2018.

[4] Musuamba FT, Skottheim Rusten I, Lesage R, Russo G, Bursi R, Emili L, et al. Scientific and regulatory evaluation of mechanistic in silico drug and disease models in drug development: Building model credibility. CPT Pharmacometrics Syst Pharmacol. 2021;10(8):804–25. 10.1002/psp4.12669 34102034 10.1002/psp4.12669 PMC8376137

[5] Elefteriades JA, Farkas EA. Thoracic aortic aneurysm clinically pertinent controversies and uncertainties. J Am Coll Cardiol. 2010;55(9):841–57. 10.1016/j.jacc.2009.08.084 20185035 10.1016/j.jacc.2009.08.084

[6] Gomez A, Wang Z, Xuan Y, Wisneski AD, Hope MD, Saloner DA, et al. Wall Stress Distribution in Bicuspid Aortic Valve-Associated Ascending Thoracic Aortic Aneurysms. Ann Thorac Surg. 2020. 10.1016/j.athoracsur.2019.12.035 32006475 10.1016/j.athoracsur.2019.12.035 PMC8598319

[7] Wang Z, Flores N, Lum M, Wisneski AD, Xuan Y, Inman J, et al. Wall stress analyses in patients with >/=5 cm versus < 5 cm ascending thoracic aortic aneurysm. J Thorac Cardiovasc Surg. 2020. 10.1016/j.jtcvs.2020.02.046 32178922 10.1016/j.jtcvs.2020.02.046 PMC8589466

[8] Cosentino F, Raffa GM, Gentile G, Agnese V, Bellavia D, Pilato M, et al. Statistical Shape Analysis of Ascending Thoracic Aortic Aneurysm: Correlation between Shape and Biomechanical Descriptors. Journal of personalized medicine. 2020;10(2). 10.3390/jpm10020028 10.3390/jpm10020028 PMC7354467 32331429

[9] Pasta S, Cannata S, Gentile G, Agnese V, Raffa GM, Pilato M, et al. Transcatheter Heart Valve Implantation in Bicuspid Patients with Self-Expanding Device. Bioengineering. 2021;8(7):91. 34356198 10.3390/bioengineering8070091 PMC8301021

[10] Azadani AN, Chitsaz S, Mannion A, Mookhoek A, Wisneski A, Guccione JM, et al. Biomechanical properties of human ascending thoracic aortic aneurysms. Ann Thorac Surg. 2013;96(1):50–8. 10.1016/j.athoracsur.2013.03.094 23731613 10.1016/j.athoracsur.2013.03.094

[11] Chung J, Lachapelle K, Cartier R, Mongrain R, Leask RL. Loss of mechanical directional dependency of the ascending aorta with severe medial degeneration. Cardiovasc Pathol. 2017;26:45–50. 10.1016/j.carpath.2016.11.001 27888778 10.1016/j.carpath.2016.11.001

[12] Shahmansouri N, Alreshidan M, Emmott A, Lachapelle K, El-Hamamsy I, Cartier R, et al. Investigation on the Regional Loss Factor and Its Anisotropy for Aortic Aneurysms. Materials (Basel). 2016;9(11). 10.3390/ma9110867 10.3390/ma9110867 PMC5457275 28773988

[13] Pasta S, Cannata S, Gentile G, Agnese V, Pilato M, Gandolfo C. Simulation of left ventricular outflow tract (LVOT) obstruction in transcatheter mitral valve-in-ring replacement. Med Eng Phys. 2020;82:40–8. 10.1016/j.medengphy.2020.05.018 32709264 10.1016/j.medengphy.2020.05.018

[14] Krishnan K, Ge L, Haraldsson H, Hope MD, Saloner DA, Guccione JM, et al. Ascending thoracic aortic aneurysm wall stress analysis using patient-specific finite element modeling of in vivo magnetic resonance imaging. Interactive cardiovascular and thoracic surgery. 2015;21(4):471–80. 10.1093/icvts/ivv186 26180089 10.1093/icvts/ivv186 PMC4627354

[15] Agnese V, Pasta S, Michelena HI, Mina C, Romano GM, Carerj S, et al. Patterns of ascending aortic dilatation and predictors of surgical replacement of the aorta: A comparison of bicuspid and tricuspid aortic valve patients over eight years of follow-up. J Mol Cell Cardiol. 2019;135:31–9. 10.1016/j.yjmcc.2019.07.010 31348923 10.1016/j.yjmcc.2019.07.010

[16] Pasta S, Phillippi JA, Gleason TG, Vorp DA. Effect of aneurysm on the mechanical dissection properties of the human ascending thoracic aorta. J Thorac Cardiovasc Surg. 2012;143(2):460–7. S0022-5223(11)00834-8 [pii] 10.1016/j.jtcvs.2011.07.058. 21868041 10.1016/j.jtcvs.2011.07.058 PMC8084112

[17] Di Giuseppe M, Alotta G, Agnese V, Bellavia D, Raffa GM, Vetri V, et al. Identification of circumferential regional heterogeneity of ascending thoracic aneurysmal aorta by biaxial mechanical testing. J Mol Cell Cardiol. 2019;130:205–15. 10.1016/j.yjmcc.2019.04.010 30998978 10.1016/j.yjmcc.2019.04.010

[18] Luraghi G, Bridio S, Miller C, Hoekstra A, Rodriguez Matas JF, Migliavacca F. Applicability analysis to evaluate credibility of an in silico thrombectomy procedure. J Biomech. 2021;126:110631. 10.1016/j.jbiomech.2021.110631 34298293 10.1016/j.jbiomech.2021.110631

[19] Ramella A, Migliavacca F, Rodriguez Matas JF, Mandigers TJ, Bissacco D, Domanin M, et al. Applicability assessment for in-silico patient-specific TEVAR procedures. J Biomech. 2023;146:111423. 10.1016/j.jbiomech.2022.111423 36584506 10.1016/j.jbiomech.2022.111423

[20] Pathmanathan P, Gray RA. Verification of computational models of cardiac electro-physiology. International journal for numerical methods in biomedical engineering. 2014;30(5):525–44. 10.1002/cnm.2615 24259465 10.1002/cnm.2615

[21] Santiago A, Butakoff C, Eguzkitza B, Gray RA, May-Newman K, Pathmanathan P, et al. Design and execution of a verification, validation, and uncertainty quantification plan for a numerical model of left ventricular flow after LVAD implantation. PLoS Comput Biol. 2022;18(6):e1010141. 10.1371/journal.pcbi.1010141 35696442 10.1371/journal.pcbi.1010141 PMC9232142

[22] Morrison TM, Hariharan P, Funkhouser CM, Afshari P, Goodin M, Horner M. Assessing Computational Model Credibility Using a Risk-Based Framework: Application to Hemolysis in Centrifugal Blood Pumps. Asaio Journal. 2019;65(4):349–60. 10.1097/Mat.0000000000000996 30973403 10.1097/MAT.0000000000000996 PMC6493688

[23] Aldieri A, Curreli C, Szyszko JA, Mattina AAL, Viceconti M. Credibility assessment of computational models according to ASME V&V40: Application to the Bologna Biomechanical Computed Tomography solution. Computer Methods and Programs in Biomedicine. 2023;240. ARTN 107727. 10.1016/j.cmpb.2023.107727. 10.1016/j.cmpb.2023.107727 37523955

[24] Elefteriades JA. Natural history of thoracic aortic aneurysms: Indications for surgery, and surgical versus nonsurgical risks. Ann Thorac Surg. 2002;74(5):S1877-S80. 12440685 10.1016/s0003-4975(02)04147-4

[25] Pasta S, Rinaudo A, Luca A, Pilato M, Scardulla C, Gleason TG, et al. Difference in hemodynamic and wall stress of ascending thoracic aortic aneurysms with bicuspid and tricuspid aortic valve. J Biomech. 2013;46(10):1729–38. 10.1016/j.jbiomech.2013.03.029 23664314 10.1016/j.jbiomech.2013.03.029 PMC4016719

[26] Galappaththige S, Gray RA, Costa CM, Niederer S, Pathmanathan P. Credibility assessment of patient-specific computational modeling using patient-specific cardiac modeling as an exemplar. Plos Computational Biology. 2022;18(10). ARTN e1010541. 10.1371/journal.pcbi.1010541. 10.1371/journal.pcbi.1010541 PMC9550052 36215228

[27] Cosentino F, Sherifova S, Sommer G, Raffa G, Pilato M, Pasta S, et al. Regional biomechanical characterization of human ascending aortic aneurysms: Microstructure and biaxial mechanical response. Acta biomaterialia. 2023;169:107–17. 10.1016/j.actbio.2023.08.016 37579911 10.1016/j.actbio.2023.08.016

[28] Farzaneh S, Trabelsi O, Avril S. Inverse identification of local stiffness across ascending thoracic aortic aneurysms. Biomechanics and modeling in mechanobiology. 2018. 10.1007/s10237-018-1073-0 30145618 10.1007/s10237-018-1073-0

[29] Azadani AN, Chitsaz S, Matthews PB, Jaussaud N, Leung J, Tsinman T, et al. Comparison of mechanical properties of human ascending aorta and aortic sinuses. Ann Thorac Surg. 2012;93(1):87–94. 10.1016/j.athoracsur.2011.08.002 22075218 10.1016/j.athoracsur.2011.08.002

