# Supplementary material for: Credibility Assessment of the Patient-Specific Modeling of the Aneurysmal Ascending Thoracic Aorta: Verification, Validation and Uncertainty Quantification
Source: Cardiovasc Eng Technol. 2025 Aug 28;16(6):663–77. doi: 10.1007/s13239-025-00801-1 (PMC12686058; doi:10.1007/s13239-025-00801-1)
Supplement: Supplementary file 1 — Supplementary material 1 [file 13239_2025_801_MOESM1_ESM.docx]

**Appendix A**

**1. Verification Activities**

Considering the verification activities and credibility factors, a detailed explanation for the grades assigned to each verification activity is given. The level of rigor achieved by the patient-specific ATAA model versus the maximum score indicated in the ASME V&V40 is presented as “credibility factor (achieved score/maximum score)”.

**1.1. Code Verification**

Software Quality Assurance (b/c): This is given a credibility factor grade b. Bugs and errors known to be present in Abaqus 2023hf2 were reviewed and assessed for the possible effects on the accuracy and credibility

Numerical Code Verification (c/d): This is given a credibility factor grade c. Discretization error was quantified by comparison to an exact solution, and a grid convergence study demonstrated that the numerical solution asymptotically approached the exact solution as the discretization was refined.

***1.2 Calculation Verification***

Discretization error (c/c): This is given a credibility factor grade c. Applicable grid or time-step convergence analyses were performed, and discretization error was estimated.

Numerical solver error (c/c): This is given a credibility factor grade c. Problem-specific sensitivity study was performed on solver parameters, confirming that changes in simulation results due to changes in the solver parameters were negligible relative to the model accuracy goal.

Use error (-/-): inputs and outputs were not verified.

**Table 1S**: summary of archived scores and maximum level of rigor along with credibility levels for verification.

| **Activity Credibility Factor** | | **Level of Rigor**  **Achieved Maximum** | | **Credibility Level** |
| --- | --- | --- | --- | --- |
| Code Verification | Software Quality Assurance | b | c | medium |
|  | Numerical Code Verification | c | d | medium-high |
| Calculation Verification | Discretization Error | c | c | high |
|  | Numerical Solver Error | c | c | high |
|  | Use Error | - | - |  |

**Validation Activities**

Considering the validation activities and credibility factors, a detailed explanation for the grades assigned to each validation activity is given in the following sub-sections. The level of rigor achieved by the patient-specific ATAA model versus the maximum score indicated in the ASME V&V40 is presented as “credibility factor (achieved score/maximum score).

***1. Computational Model***

***1.1 Model Form***

Model Form (b/c): This is given a credibility factor grade b. The influence of expected key model form assumptions was explored. During model development, shell modelling with uniform thickness was considered. Shell model appeared robust in reducing element distortion and improving computational efficiency.

***1.2 Model Input***

Quantification of Sensitivities (b/c): This is given a credibility factor grade b. A sensitivity analysis was performed only a limited set of parameters, based on engineering judgement. As a matter of fact, exploring the complete set of parameters (which assessment is not straightforward) was not deemed impractical due to the excessively high computational demand.

Quantification of Uncertainties (b/c): This is given a credibility factor grade b. Similarly, to the quantification of sensitivities, uncertainties were identified and quantified only for key input parameters.

***2. Comparator***

***2.1 Test Samples (Patients)***

Quantity (b/c): This is given a credibility factor grade b as multiple samples were used, but not enough to be statistically relevant. For instance, the six patient-specific ATAA models does not allows to perform statistical comparison to assess the relevance of aortic valve (BAV vs TAV) on model output.

Range of Characteristics (d/d): This is given a credibility factor grade b as samples represents the entire range of parameters. The investigated patient-specific ATAA models covered the entire range of patients with ATAAs.

Measurements of Test Samples (b/c): This is given a credibility factor grade b as one key characteristics of the test samples (ie, the aortic diameter) was measured.

Uncertainty of Measurements (c/d): This is given a credibility factor grade c as the uncertainty analysis incorporated instrument accuracy and repeatability in the aortic diameter measurements for all patients. Inter-operator variability on aortic diameter measurements was not evaluated.

***2.2 Test (Patient) Conditions***

Quantity of test condition (a/c): This is given a credibility factor grade a as one patient condition at rest was examined.

Range of test condition (a/d): This is given a credibility factor grade a single test condition at rest was examined.

Measurements of test conditions (c/c): This is given a credibility factor grade c. Though one key characteristics of the test (patient) condition (ie, the aortic diameter) was measured, three measurements were done for each patient at different anatomic levels.

Uncertainty of Measurements (c/d): This is given a credibility factor grade c as the uncertainty analysis incorporated instrument accuracy and repeatability in the aortic diameter measurements. Other conditions such as the segmentation uncertainty was not evaluated.

***3. Assessment***

***3.1 Equivalency of Input Parameters***

Equivalency of Input Parameters (c/c): This is given a credibility factor grade c as the types and ranges of all inputs were equivalent.

***3.2 Output Comparison***

Quantity (b/b): This is given a credibility factor grade b as multiple outputs were compared.

Equivalence of Output Parameters (c/c): This is given a credibility factor grade c as the types of all outputs were equivalent.

Rigor of Comparison (d/d): This is given a credibility factor grade d as the uncertainties both in the computational model and the comparator were included in the comparison.

Agreement of Comparison (b/c): This is given a credibility factor grade b as the level of agreement of the output comparison was satisfactory for key comparisons, but not all comparisons.

**4. Applicability of the Validation Activities to the COU**

Relevance of QoI (b/c): This is given a credibility factor grade b as a subset of the QoI from the validation activities were identical to those for the COU. The biomechanical behavior of patient-specific ATAA model matched the aortic diameter response in four out of six patient cases.

Relevance of the Validation Activities to the COU (c/d): This is given a credibility factor grade c as the COU encompassed some of the validation points in four out of six patients.

**Table 2S**: summary of archived scores and maximum level of rigor along with credibility levels for validation and applicability assessment

| **Activity Credibility Factor** | | **Level of Rigor**  **Achieved Required** | | **Credibility Level** |
| --- | --- | --- | --- | --- |
| Computational Model | Model Form | b | c | medium |
|  | Model Input  Quantification of Sensitivities | b | c | medium |
|  | Quantification of Uncertainties | b | c | medium |
| Comparator | Test Samples  Quantity | b | c | medium |
|  | Range of Characteristics | d | d | high |
|  | Measurements | b | c | medium |
|  | Uncertainty of Measurements | c | d | medium-high |
|  | Test Conditions  Quantity | a | c | low |
|  | Range | a | d | low |
|  | Measurements | c | c | high |
|  | Uncertainty of Measurements | c | d | medium-high |
| Assessment | Equivalency of Input Parameters | c | c | high |
|  | Output Comparison  Quantity | b | b | high |
|  | Equivalence of Output Parameters | c | c | high |
|  | Rigor of Comparison | d | d | high |
|  | Agreement of Comparison | b | c | medium |
| **Applicability of the Validation Activities to the COU** | |  |  |  |
| Relevance of the QOIs | | b | c | medium |
| Relevance of the Validation Activities to the COU | | c | d | medium-high |
